# Flexural Strength of Different Commercially Available Auto-Polymerizing Acrylic Resins: An In Vitro Study

**DOI:** 10.7759/cureus.71905

**Published:** 2024-10-20

**Authors:** Soumalya Banerjee, Shruti Jha, Rupa Singh, Harendra Shahi, Aishwarya Arya, Rahul Raj

**Affiliations:** 1 Prosthodontics, Crown and Bridge, Mithila Minority Dental College and Hospital, Darbhanga, IND; 2 Prosthodontics, Crown and Bridge, Banerjee Dental Clinic, Bihar, IND; 3 Conservative Dentistry and Endodontics, Awadh Dental College and Hospital, Jamshedpur, IND; 4 Prosthodontics, Crown and Bridge, Oxford Dental College and Hospital, Bangalore, IND

**Keywords:** auto-polymerizing acrylic resins, dental materials, dpi, flexural strength, pyrax, quick ashwin, three-point bending test

## Abstract

Background

Auto-polymerizing acrylic resins, composed primarily of poly(methyl methacrylate) and methyl methacrylate, are crucial in prosthetic dentistry due to their ease of use, cost-effectiveness, and acceptable aesthetics, polymerizing at room temperature to form solid polymer networks. One of the most important mechanical properties of these resins is flexural strength, which is essential for their performance under continuous masticatory forces. In clinical applications, prosthetic materials endure repetitive stress from chewing, and resins with higher flexural strength are better equipped to resist deformation and fractures. This property is vital for ensuring the long-term integrity of prostheses, minimizing the risk of material failure, and enhancing prosthetic longevity, thus contributing to better clinical outcomes and patient satisfaction.

Objective

Resins with superior flexural strength, such as DPI, provide better clinical outcomes. High flexural strength minimizes the risk of material failure in high-stress areas, especially in extensive restorations like temporary bridges or denture repairs. For clinicians, using materials with greater strength reduces the likelihood of restoration failure, ensuring more reliable performance and fewer interventions for repair or replacement. This study aims to evaluate and compare the flexural strength of three commercially available auto-polymerizing acrylic resins, Pyrax (Pyrax International, Uttarakhand, India), DPI (Dental Products of India, Mumbai, India), and Quick Ashvin (National Dental Supply Company, Delhi, India), to determine their suitability for various dental applications.

Methodology

Following American Dental Association specifications, 20 rectangular bar-shaped specimens (25 mm x 2 mm x 2 mm) of each resin type were prepared. The resins were mixed according to manufacturer instructions, poured into molds, and allowed to auto-polymerize at room temperature. Post-polymerization, specimens were immersed in artificial saliva at 37°C for 10 days to simulate oral conditions. Flexural strength was assessed using a three-point bending test on a universal testing machine, and results were analyzed using ANOVA and post-hoc Bonferroni tests.

Results

The flexural strength of DPI resin bars (mean=482.10 MPa, SD=17.28) was significantly higher compared to both Pyrax (mean=467.82 MPa, SD=15.01) and Quick Ashvin (mean=470.17 MPa, SD=8.18). ANOVA revealed a significant difference among the resins (F=5.956, p=0.004). Post-hoc Bonferroni comparisons showed a statistically highly significant difference between DPI and Pyrax (p=0.006) and a significant difference between DPI and Quick Ashvin (p=0.028). No significant difference was observed between Pyrax and Quick Ashvin (p=1.000).

Conclusion

DPI resin, with its superior flexural strength, is the best choice for high-stress dental applications, while Quick Ashvin offers balanced performance for interim uses such as temporary crowns. Pyrax, being more cost-effective, is suitable for less demanding cases. This study underscores the importance of selecting materials based on mechanical properties and calls for further research to enhance resin durability. For dental practitioners, DPI is ideal for long-lasting restorations, Quick Ashvin for temporary solutions, and Pyrax for budget-conscious cases.

## Introduction

Auto-polymerizing acrylic resins are widely used in prosthetic dentistry for their ease of manipulation, cost-effectiveness, and acceptable aesthetics. These materials, composed of poly(methyl methacrylate) polymer and methyl methacrylate monomer, undergo a polymerization process at room temperature, forming a solid polymer network. The mechanical properties of these resins, particularly flexural strength, are crucial to their performance in dental applications [1]. Flexural strength, which measures a material's resistance to deformation under load, is particularly important for prostheses subjected to masticatory forces, as it directly impacts their clinical durability [2,3].

Flexural strength directly influences the longevity of prosthetics, especially in the oral environment where chewing forces are significant. Higher flexural strength means less susceptibility to cracking or breaking, contributing to longer-lasting prostheses and improved patient satisfaction. Current knowledge gaps, particularly in the performance of auto-polymerizing resins under varied clinical conditions, underscore the need for more studies evaluating long-term wear resistance, biocompatibility, and real-world performance [3].

The polymerization process and chemical composition of auto-polymerizing acrylic resins, including the presence of cross-linking agents, significantly influence their mechanical properties. Materials with higher degrees of polymerization and cross-linking tend to exhibit superior flexural strength and resistance to fracture. Additives such as glass fibers or reinforcing agents can further enhance these properties, although their type and amount must be controlled to avoid compromising the resin's biocompatibility and aesthetics. Studies comparing the flexural strength of different commercially available resins provide valuable insights into their suitability for various dental applications [4].

In a comparative evaluation of three widely used auto-polymerizing acrylic resins, Pyrax (Pyrax International, Uttarakhand, India), DPI (Dental Products of India, Mumbai, India), and Quick Ashvin (National Dental Supply Company, Delhi, India), DPI resin demonstrated the highest flexural strength, followed by Quick Ashvin and Pyrax. The superior performance of DPI resin is attributed to its optimized chemical composition and cross-linking agents, enhancing its resistance to deformation. Conversely, Pyrax resin exhibited the lowest flexural strength, suggesting limited suitability for applications requiring high mechanical resilience. These findings underscore the importance of selecting materials with high flexural strength to ensure the long-term success of dental restorations subjected to significant mechanical stress.

## Materials and methods

Specimen preparation and testing protocol

Resin Specimen Preparation

The preparation of resin specimens was carried out following the American Dental Association (ADA) specifications, specifically designed to evaluate the mechanical properties of dental materials. Each resin's powder and liquid components were carefully mixed in accordance with the respective manufacturer's instructions. This step ensured that each resin was prepared consistently, avoiding variability that could affect the mechanical properties.

Once the components were properly mixed, the mixtures were poured into prefabricated molds designed to create rectangular specimens measuring 25 mm x 2 mm x 2 mm, following the ADA guidelines for standard testing of materials. The dimensions were chosen based on recommendations for conducting three-point bending tests, an established method for measuring the flexural strength of dental resins.

The poured resin mixtures were allowed to auto-polymerize at room temperature. This process was essential to ensure the proper curing of the resin without external heat or light, as auto-polymerizing resins set naturally at room temperature, mimicking clinical conditions.

**Figure 1:**
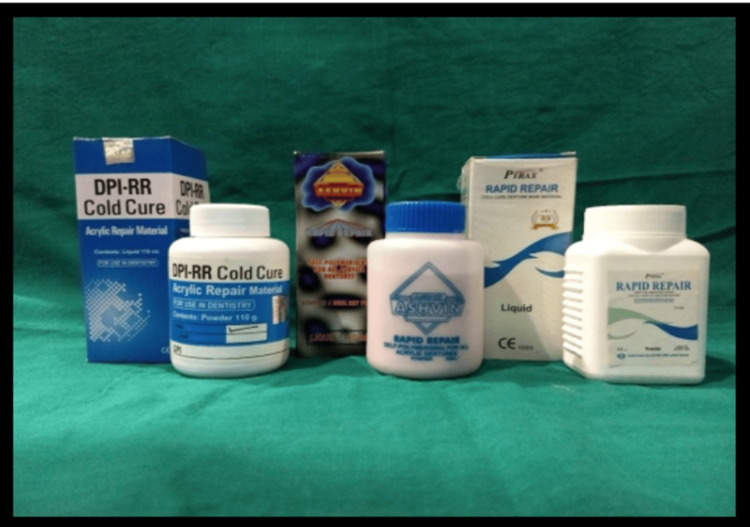
Auto-polymerizing resins used in the study

After polymerization, the specimens were carefully removed from the molds and immediately immersed in artificial saliva at 37°C for a duration of 10 days. This step simulated the moist environment of the oral cavity, where these materials are typically used. The 10-day immersion allowed the specimens to stabilize and mimic the clinical aging of dental resins before mechanical testing. This method helps in evaluating the real-world behavior of the resins, ensuring that the mechanical properties tested are reflective of the in vivo environment (Figure 1) [4].

**Figure 2:**
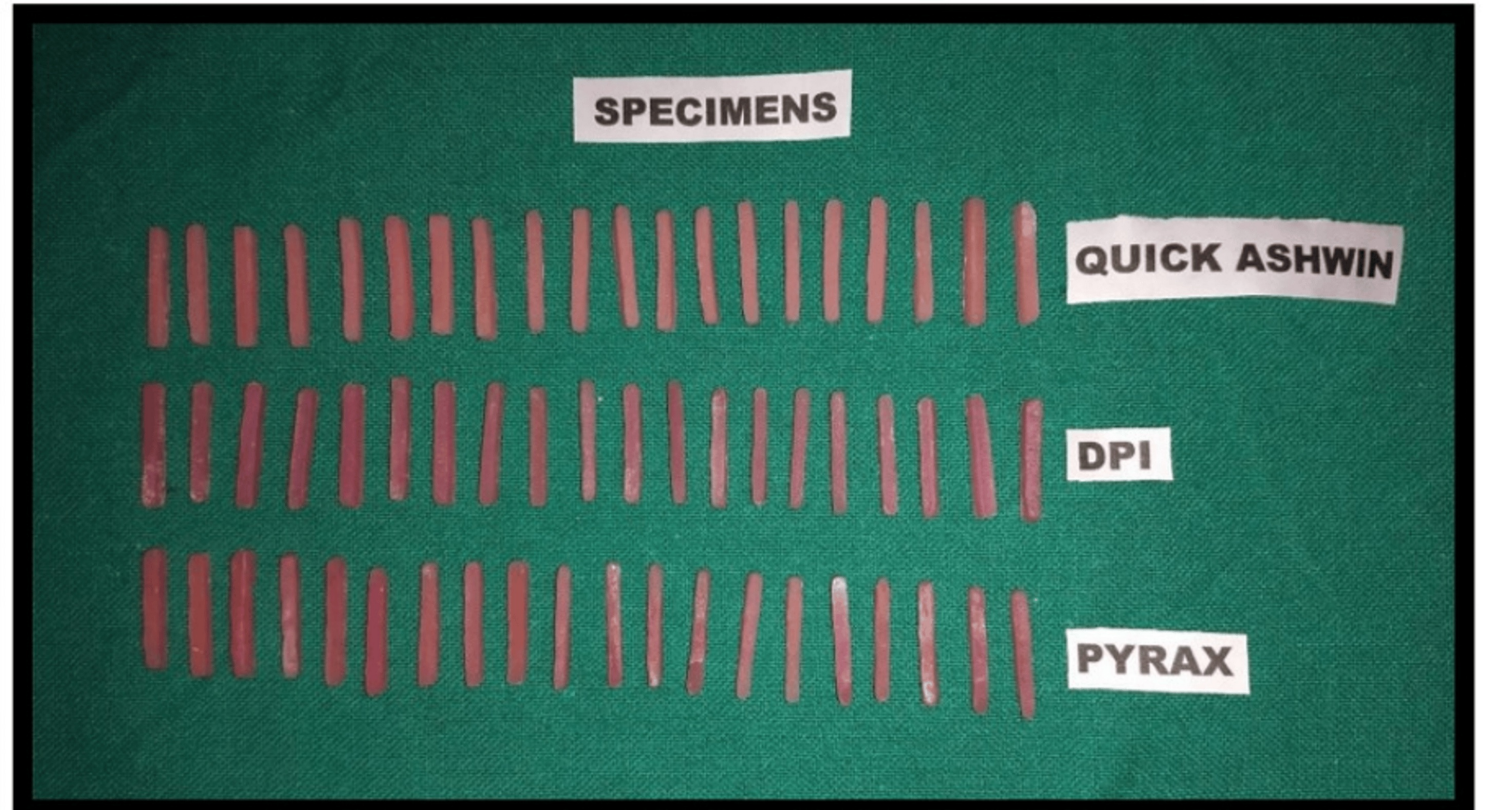
Pyrax, DPI, and Quick Ashvin study samples

The mixtures were poured into prefabricated molds to form rectangular specimens, each measuring 25 mm x 2 mm x 2 mm, in accordance with ADA guidelines for three-point bending tests. Once poured, the molds were left at room temperature to allow the resins to auto-polymerize (Figure 2).

After polymerization, the specimens were carefully removed from the molds and immersed in artificial saliva at 37°C for 10 days. This step was crucial to simulate the moist environment of the oral cavity, ensuring that the specimens would be exposed to conditions similar to those encountered during actual clinical use. The 10-day storage period allowed the resins to reach a stable state before undergoing mechanical testing.

Three-Point Bending Test

**Figure 3:**
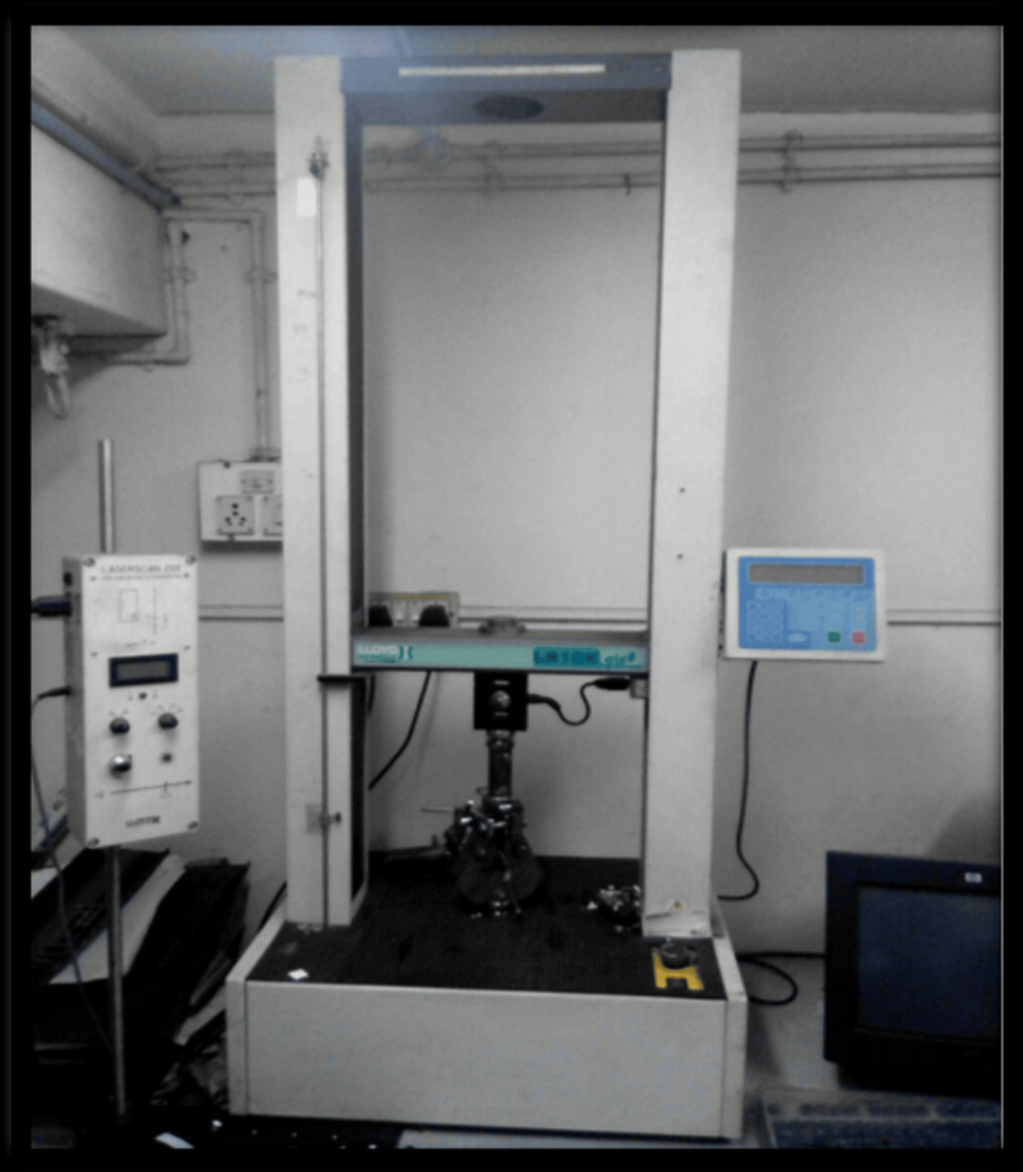
Universal testing machine - Lloyd Instruments Lr 10k Plus

The three-point bending test is a widely accepted method for evaluating the flexural strength of dental materials. In this study, the flexural strength of each resin specimen was assessed using a universal testing machine, specifically the Lloyd Instruments Lr 10k Plus (AMETEK, Hampshire, UK) (Figure 3).

The resin specimens were placed on two supports with a 20-mm span between them, with the force applied at the midpoint of the specimen. The crosshead of the machine was set to move at a constant speed of 0.75 mm/min, in compliance with the ISO 4049 standard, which governs the mechanical testing of dental materials.

As the load was applied, the specimen gradually bent until it fractured. The force required to induce fracture was recorded in Newtons (N). This force represents the material's resistance to bending and is crucial in determining its ability to withstand occlusal forces in the oral cavity (Figure 3).

The flexural strength (σf) of each specimen was calculated using the following formula:

σf​=3FL/2bd 2​

where σf is the flexural strength, F is the fracture load, L is the span length (20 mm), b is the width of the specimen, and d is the thickness of the specimen.

Statistical analysis

The data collected from the three-point bending tests were subjected to statistical analysis using SPSS Statistics version 26 (IBM Corp. Released 2019. IBM SPSS Statistics for Windows, Version 26.0. Armonk, NY: IBM Corp.) to compare the flexural strength of Pyrax, DPI, and Quick Ashvin resins. An ANOVA was performed to determine whether there were significant differences in flexural strength among the three materials.

## Results

A total of 60 bar specimens made from three different commercially available auto-polymerizing acrylic resins (20 each from Pyrax, DPI, and Quick Ashvin) were tested for flexural strength. The results demonstrate a significant variation in flexural strength among the three resins.

Comparative flexural strength analysis

Bars made from DPI resin exhibited the highest flexural strength compared to Pyrax and Quick Ashvin resins. The mean flexural strength of DPI resin bars was 482.10 MPa (SD=17.28), followed by Quick Ashvin at 470.17 MPa (SD=8.18) and Pyrax at 467.82 MPa (SD=15.01).

**Figure 4:**
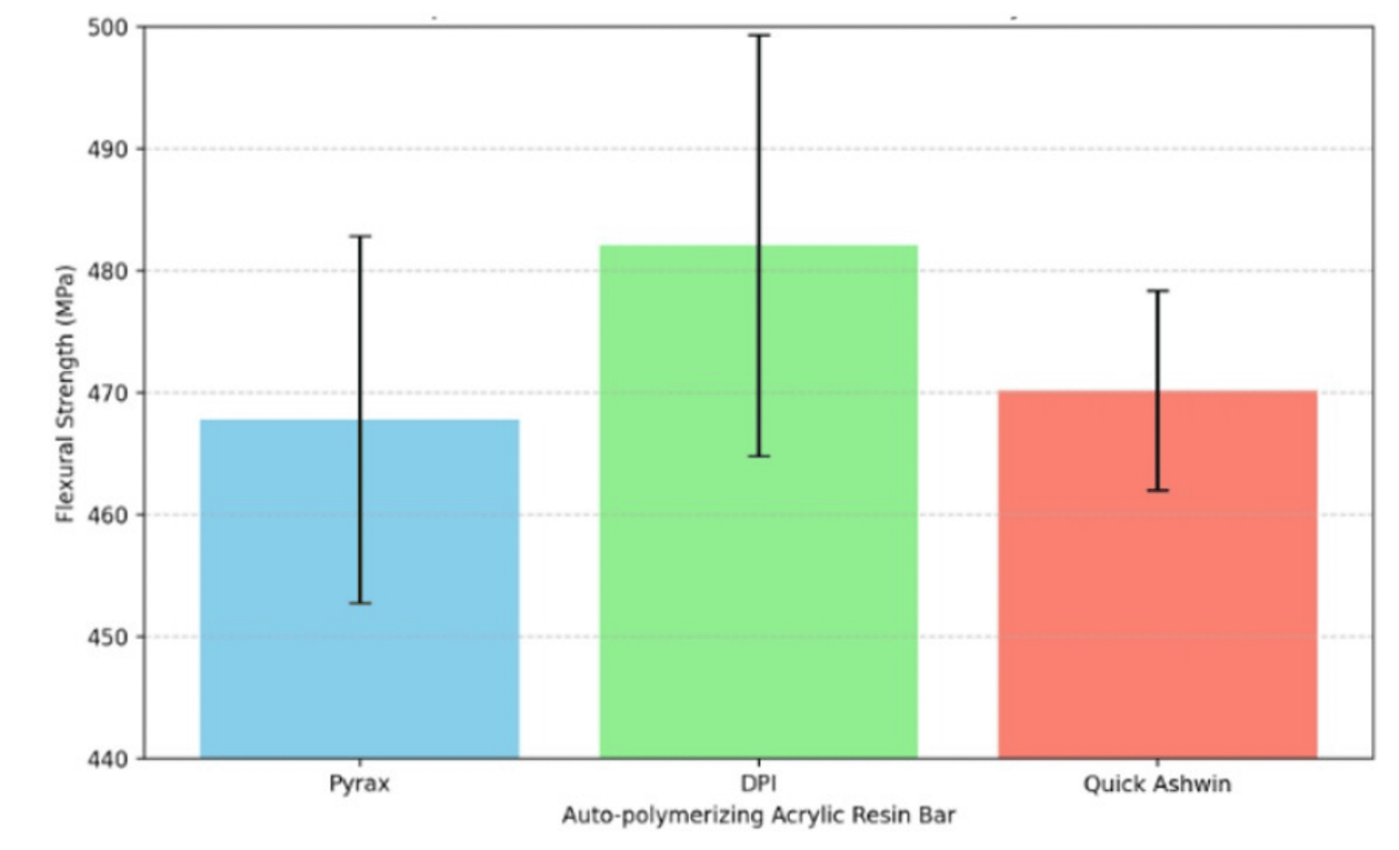
Comparison of flexural strength of different acrylic variables

Statistical analysis using ANOVA revealed a significant difference in flexural strength among the three groups of resins (F=5.956, p=0.004) (Table 1, Figure 4). Further post-hoc Bonferroni comparisons provided detailed insights into these differences. The flexural strength between DPI and Pyrax was found to be statistically highly significant, with a p-value of 0.006. Similarly, the difference between DPI and Quick Ashvin was statistically significant, with a p-value of 0.028. However, no statistically significant difference was observed between Pyrax and Quick Ashvin, as indicated by a p-value of 1.000.

**Table 1:** Comparison of flexural strength of bars made from different commercially available auto polymerizing acrylic resins SD: standard deviation, CI: confidence interval ANOVA (F) = 5.956, p=0.004 (highly significant)

Auto-polymerizing acrylic resin bar	n	Flexural strength	95% CI for mean		Minimum	Maximum
		Mean ± SD	Lower	Upper		
Pyrax	20	467.82 ± 15.00	460.7997	474.8483	443.32	493.28
DPI	20	482.10 ±17.27	474.0185	490.1895	456.83	519.41
Quick Ashvin	20	470.16 ±8.18	466.3395	473.9997	454.20	484.06
Total	60	473.36 ±15.16	469.4483	477.2835	443.32	519.41

Post-hoc Bonferroni tests were then conducted to identify specific pairs of resins that exhibited statistically significant differences in their mechanical performance (Table 2).

**Table 2:** Multiple comparisons using post-hoc Bonferroni test

Groups	p-value
Pyrax	0.006 (highly significant)
Pyrax vs, Quick Ashvin	1.000 (not significant)
DPI	0.028 (significant)

In terms of range and confidence intervals, the flexural strength for Pyrax ranged from 443.32 MPa to 493.28 MPa, with a 95% confidence interval of 460.80 to 474.85 MPa. DPI resin exhibited flexural strength ranging from 456.83 MPa to 519.41 MPa, with a 95% confidence interval of 474.02 to 490.19 MPa. Quick Ashvin resin's flexural strength was recorded between 454.20 MPa and 484.06 MPa, with a 95% confidence interval of 466.34 to 474.00 MPa.

Effect size (Cohen's d)

**Figure 5:**
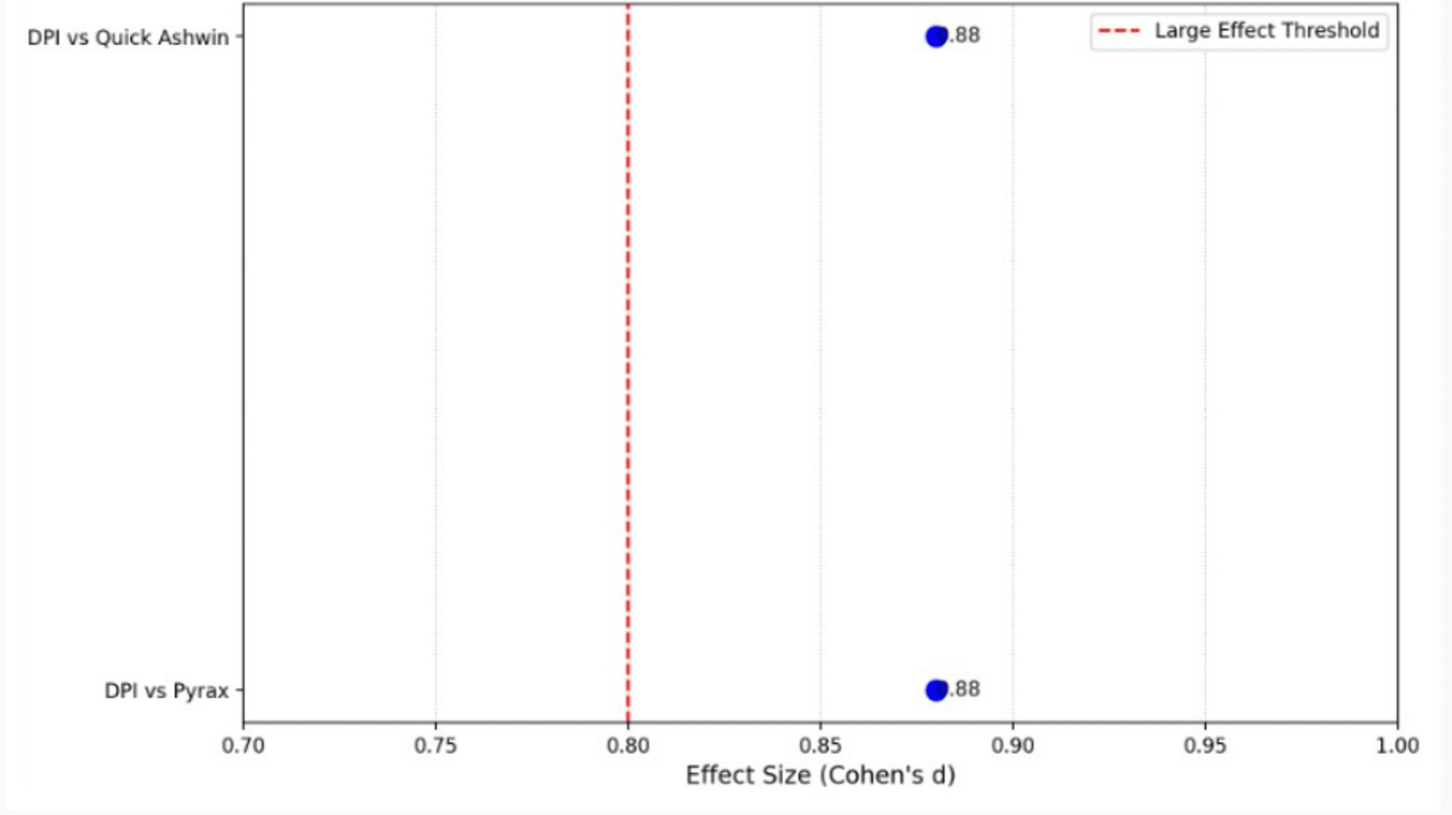
Dot plot showing the effect size threshold using Cohen's d test

The effect sizes (Cohen's d) provide a measure of the magnitude of differences in flexural strength between the groups. Cohen's d values of 0.2 are considered small, 0.5 medium, and 0.8 or greater large. For the significant comparisons in this study, the calculated effect sizes show large effects. Specifically, the difference in flexural strength between DPI and Pyrax resulted in a Cohen's d of 0.88, and the comparison between DPI and Quick Ashvin also yielded a Cohen's d of 0.88. These values indicate that the differences in flexural strength between DPI and both Pyrax and Quick Ashvin are not only statistically significant but also substantial, with a large effect size in both cases. This suggests that DPI is considerably stronger than the other two resins in terms of mechanical performance (Figure 5).

The results indicate that DPI resin consistently exhibited significantly higher flexural strength compared to both Pyrax and Quick Ashvin, making it the most mechanically robust material in this study. However, the comparison between Pyrax and Quick Ashvin showed no significant difference in their flexural strength, indicating that their mechanical performance is quite similar under the tested conditions.

## Discussion

The findings of this study align with existing research, underscoring the significance of material composition and polymerization techniques in determining the flexural strength of auto-polymerizing acrylic resins. The superior performance of DPI resin stands out, reflecting its robust chemical structure and polymerization properties. DPI resin's high flexural strength is primarily attributed to its advanced chemical composition, which includes effective cross-linking agents and hydrophobic properties [5]. Cross-linking agents are crucial as they form a more interconnected and rigid polymer network, enhancing the material's resistance to deformation and fracture [6]. This finding supports previous research that demonstrated the positive impact of cross-linking agents on the mechanical properties of dental resins. Additionally, the hydrophobic characteristics of DPI resin reduce water absorption, thereby limiting plasticization and maintaining structural integrity in the moist oral environment, a vital factor for materials used in dental restorations.

Polymerization techniques also play a pivotal role in the mechanical performance of these resins. The rapid polymerization process of DPI resin creates a more uniform polymer network, contributing to its superior mechanical properties [7,8]. Faster polymerization rates, as shown in prior studies, are linked to enhanced material strength and stability, which was reflected in the consistently higher flexural strength values of DPI in this study. Furthermore, the thermal stability of DPI resin ensures that it retains its mechanical performance under the stress conditions applied during testing, further contributing to its robustness [9].

In comparison, Quick Ashvin resin, while not as strong as DPI, demonstrated moderate flexural strength due to the presence of cross-linking agents and hydrophobic features, albeit less effective than those found in DPI [10]. Pyrax resin exhibited the lowest flexural strength, which can be attributed to its less robust chemical composition, fewer cross-linking agents, and reduced hydrophobicity, making it less suitable for high-stress applications [10,11]. These variations in performance highlight the importance of selecting appropriate materials based on the specific mechanical demands of the clinical situation [12,13].

Clinically, the selection of resins with higher flexural strength, such as DPI, is crucial for applications subjected to high mechanical stress, such as long-span interim prostheses or the repair of fractured dental appliances [14]. The enhanced durability of DPI resin can reduce the risk of fracture, extending the lifespan of restorations and ultimately benefiting patients by minimizing the need for frequent repairs [15,16]. Quick Ashvin, with its moderate strength and cost-effectiveness, serves as a viable option for less demanding applications, while Pyrax, being the least robust, is better suited for cost-sensitive cases where mechanical strength is not the primary concern.

The study's findings are consistent with previous research on the mechanical properties of auto-polymerizing acrylic resins. Various studies have similarly reported that resins with improved chemical compositions, particularly those featuring effective cross-linking and hydrophobic agents, exhibit superior mechanical properties. The consistency of these results with existing literature strengthens the credibility of the conclusion that DPI resin is the most suitable material for high-strength applications in dental practice [17].

However, certain limitations of the study should be acknowledged. The research was conducted under controlled laboratory conditions, which may not fully replicate the dynamic oral environment, where factors such as temperature fluctuations, humidity, and variable mechanical loads could influence material performance. Additionally, this study focused solely on flexural strength without evaluating other important properties such as biocompatibility, wear resistance, and color stability, which are also critical for clinical success. Future research should address these limitations by conducting in vivo studies and exploring a broader range of material properties to provide a more comprehensive understanding of these resins' long-term performance in dental restorations.

Future perspectives

For clinicians, the main takeaway is to select materials based on the mechanical demands of the case. DPI is recommended for high-stress environments due to its superior strength, while Quick Ashvin is suitable for interim or less demanding applications. Pyrax should be reserved for cost-sensitive cases where lower strength is acceptable. Future research should expand on these findings by evaluating other mechanical properties such as wear resistance, biocompatibility, and long-term performance under varying environmental conditions, including temperature and humidity changes.

## Conclusions

The comparative evaluation of DPI, Quick Ashvin, and Pyrax auto-polymerizing acrylic resins offers valuable insights into their mechanical properties and their appropriateness for various dental applications. DPI resin demonstrated superior performance, making it the ideal choice for high-stress applications, attributed to its enhanced flexural strength and advanced chemical composition. Quick Ashvin, with its balanced performance, is well-suited for a range of interim dental uses, while Pyrax provides a cost-effective solution for less demanding cases. This study emphasizes the critical role of material selection in clinical practice and highlights the need for continued research aimed at improving the performance and durability of dental resins. By further investigating the interplay between chemical composition, polymerization methods, and mechanical properties, the dental industry can develop materials that better address the demands of modern practice and improve patient outcomes.
